# Supplementary material for: Prevalence and associated factors of post-partum depression in Ethiopia. A systematic review and meta-analysis
Source: PLoS One. 2021 Feb 19;16(2):e0247005. doi: 10.1371/journal.pone.0247005 (PMC7894900; doi:10.1371/journal.pone.0247005)
Supplement: S1 File — (DOCX) [file pone.0247005.s002.docx]

The pooled prevalence of postpartum depression among women after childbirth in the community and in the health-institution were 20.42% with 95% CI (15.06, 25.79) and 24.15% with 95% CI 18.53, 29.78) respectively. This showed the prevalence in both health institution and community is similar. To build the graph, prevalence of PPD, standard error of each prevalence, the site (health institution and community-based studies), year of publication, authors’ name, and 95% CI were included. The detail is in the figure below.

NOTE: Weights are from random effects analysis

.

.

Overall (I-squared = 95.1%, p ≤ 0.001)

Melkamu M. (2020)

Kerie S. (2018)

Shitu S. (2019)

ID

Abebe A. (2019)

Teshome H (2016)

Subtotal (I-squared = 88.0%, p ≤ 0.001)

Shewangzaw A. (2018)

Subtotal (I-squared = 95.1%, p ≤ 0.001)

H/Mariam D. (2016)

Study

community based

health institution

Azale T (2018)

Fantahun A. (2018)

Abadiga M. (2019)

Toru T. (2018)

22.08 (17.66, 26.49)

25.00 (21.30, 28.70)

33.80 (29.21, 38.39)

23.70 (20.29, 27.11)

ES (95% CI)

22.10 (18.50, 25.70)

27.80 (22.69, 32.91)

24.15 (18.53, 29.78)

13.11 (7.12, 19.10)

20.42 (15.06, 25.79)

19.00 (15.90, 22.10)

12.20 (11.06, 13.34)

23.30 (19.97, 26.63)

20.90 (16.20, 25.60)

22.40 (18.57, 26.23)

100.00

9.19

8.88

9.28

Weight

9.22

8.68

44.41

8.32

55.59

9.37

%

9.76

9.30

8.84

9.15

22.08 (17.66, 26.49)

25.00 (21.30, 28.70)

33.80 (29.21, 38.39)

23.70 (20.29, 27.11)

ES (95% CI)

22.10 (18.50, 25.70)

27.80 (22.69, 32.91)

24.15 (18.53, 29.78)

13.11 (7.12, 19.10)

20.42 (15.06, 25.79)

19.00 (15.90, 22.10)

12.20 (11.06, 13.34)

23.30 (19.97, 26.63)

20.90 (16.20, 25.60)

22.40 (18.57, 26.23)

100.00

9.19

8.88

9.28

Weight

9.22

8.68

44.41

8.32

55.59

9.37

%

9.76

9.30

8.84

9.15

0

25

50

75

100
